# Feasibility of De-Escalation Implementation for Positive Blood Cultures in Patients With Sepsis: A Prospective Cohort Study

**DOI:** 10.3389/fphar.2020.576849

**Published:** 2021-02-12

**Authors:** José Victor de Miranda Pedroso, Fabiane Raquel Motter, Sonia Tiemi Koba, Mayara Costa Camargo, Maria Inês de Toledo, Fernando de Sá Del Fiol, Marcus Tolentino Silva, Luciane Cruz Lopes

**Affiliations:** ^1^ Postgraduate Program in Pharmaceutical Sciences, University of Sorocaba, UNISO, São Paulo, Brazil; ^2^ Posgraduate Program in Tropical Medicine, University of Brasilia (UnB), Brasília, Brazil

**Keywords:** sepsis, antimicrobial stewardship, antimicrobial drug resistance, intensive care units, anti-bacterial agents [MeSH]

## Abstract

**Purpose:** The aim of the present study was to determine whether de-escalation guided by blood cultures for patients with a diagnosis of sepsis, severe sepsis or septic shock reduces mortality, and antimicrobial drug resistance (ADR).

**Methods: **A prospective, single-center, cohort study was conducted with adults admitted to the ICU with a diagnosis of sepsis, severe sepsis, or septic shock at a public hospital in Sorocaba, State of São Paulo, Brazil, from January 2013 to December 2013. We excluded patients who had negative blood cultures. Patients who had replaced the initial empirical broad-spectrum antibiotic therapy (EAT) by the antibiotic therapy guided by blood cultures were compared with those who continued receiving EAT. The outcome included mortality and antimicrobial drug resistance. We used the Cox regression (proportional hazards regression) and the Poisson regression to analyze the association between antibiotic therapy guided by blood cultures (ATGBC) and outcomes. The statistical adjustment in all models included the following variables: sex, age, APACHE II (Acute Physiology And Chronic Health Evaluation II) score and SOFA (Sequential Organ Failure Assessment) score.

**Results: **Among the 686 patients who were admitted to the intensive care unit, 91 were included in this study. The mean age of the patients was 52.7 years (standard deviation = 18.5 years) and 70.3% were male. EAT was replaced by ATGBC in 33 patients (36.3%) while 58 patients (63.7%) continued receiving EAT. Overall hospital mortality decreased from 56.9% in patients who received EAT to 48.5% in patients who received ATGBC [Hazard ratio- HR 0.44 (95% CI 0.24–0.82), *p* = 0.009]. There was no association between ATGBC and ADR [HR 0.90 (95% CI 0.78 – 1.03) *p* = 0.15].

**Conclusions: **Although the early and appropriate empirical EAT is undoubtedly an important factor prognostic, ATGBC can reduce the mortality in these patients.

## Introduction

Sepsis remains a serious public health problem with high morbidity and mortality. In 2017, the most recent global estimates for sepsis incidence reported 48, 9 million incident cases of sepsis and 11 million sepsis-related deaths, representing 19.7% of all global deaths (Rudd et al., 2020). Studies based on data for adults admitted to hospital show that mortality rates of sepsis range from 26.4% to 55.7% (Vincent et al., 2006; Engel et al., 2007; Machado et al., 2017; Strandberg et al., 2020; Xie et al., 2020), contributing to one-third to half of the deaths of hospitalized patients. Although therapeutic measures with considerable positive impacts have been widely emphasized, the management of sepsis in critically ill patients is challenging.

Empirical broad-spectrum antibiotic therapy (EAT) for treating sepsis, severe sepsis, and septic shock, when appropriate, reduces mortality; however, there is a risk that this treatment may expose patients to the overuse of antibiotics. It can lead to increases in antibiotic resistance, costs, and drug-associated adverse events (Martínez et al., 2020; Rhee et al., 2020). Thus, studies investigating the association between EAT and mortality among patients with sepsis have reported different findings (Paul et al., 2010). In addition, there is a relatively small number of studies that directly address the impact of the appropriate selection of antibiotic therapy in these patients (Sherwin et al., 2017).

One of the main concerns on the management of patients with sepsis is the increase in the number of infections due to multidrug-resistant pathogens which limits the treatment options. Strategies focusing on the rational use of antibiotics are essential to ensure successful outcomes and to prevent adverse antibiotic effects, and the spread of antimicrobial drug resistance (ADR) (Pradipta et al., 2013; Denny et al., 2019). In this context, antibiotic therapy guided by blood culture (ATGBC) has been proposed as a strategy to reduce unnecessary exposure to antibiotics. Even if it appears beneficial, the impact of this strategy on mortality outcomes are uncertain (Lambregts et al., 2019).

In light of these uncertainties, investigating and improving the management of antimicrobial therapy in critically ill patients with sepsis is crucial to ensure high quality and safe patient care (Mathur, 2019). Moreover, there is limited data on the impact of these strategies in patients with sepsis in low and middle-income countries such as Brazil. Therefore, this study aims to determine whether antibiotic therapy guided by blood cultures for adult patients with a diagnosis of sepsis, severe sepsis, or septic shock reduces mortality and length of hospital stay, considering relevant factors such as sex, age, and severity of sepsis.

## Methods

### Study Design

A prospective, single-center, cohort study was conducted to compare mortality and length of stay in a hospital between patients who have had initial empiric therapy replaced by blood culture-guided therapy and those patients who continue receiving EAT for treatment of sepsis or sepsis severe at a public hospital in Sorocaba, State of São Paulo, Brazil, from January 2013 to December 2013. This study was registered at Brazilian Clinical Trials Registry (ReBEC number 98.772, UTN: U1111–1,142–1806) and it was performed in accordance with the Strengthening the Reporting of Observational Studies in Epidemiology (STROBE) guidelines (Von Elm et al., 2014).

### Setting

This study was carried out in the Intensive Care Unit (ICU) of the Conjunto Hospitalar de Sorocaba (CHS), Brazil. This public hospital is part of the fourth administrative region of the State of São Paulo (Regional Health Division XVI), which serves around three million inhabitants from 48 municipalities. The CHS is a tertiary university hospital with 28 beds destined to the ICU where patients are treated by a multidisciplinary healthcare team including 30 physicians and 20 residents in clinical and surgical clinical, the technical nurse responsible and 60 nursing technicians. It is also a hospital qualified as a Teaching Institution supported by the Faculty of Medical and Health Sciences of the Pontifical Catholic University of São Paulo.

### Study Population

Study subjects included hospitalized adult patients (aged ≥18 years) with a diagnosis of sepsis, severe sepsis, or septic shock according to American College of Chest Physicians (ACCP) infection criteria, who were admitted to the ICU from January 2013 to December 2013. All patients who had positive results in at least two blood culture were included in order to avoid errors related to colonization/contamination of surgical sites.

### Definitions

Blood samples, secretion cultures (according to their origin), and blood cultures (at least three different sites) were collected for routine admission exams according to the protocols adopted by the CHS.

Blood cultures were performed by an automated process using the Bact-Alert system manufactured by bioMérieux, Inc (100 Rudolph Wtreet, Durham, NC 27712).

EAT was defined as an initial antibiotic treatment before the microorganism was identified and antimicrobial susceptibility test results were obtained. EAT was initiated for all patients included. After the result of blood cultures, the subjects were classified into two groups, according to clinical evolutionary criteria (Bone et al., 1992). Patients who were progressing satisfactorily remained on broad-spectrum EAT and those who did not show clinical improvement had their therapy replaced by an antibiotic therapy guided by blood culture which was based on blood culture and antibiotic susceptibility results.

Clinical and laboratory follow-up was carried out by attending physicians and infectious disease specialists, with an adaptation to the most specific antimicrobial therapy (smaller or larger generation), if possible for monotherapy and a shorter period of therapy.

### Data Collection

We developed a software in the Access platform in order to collect data from patients included in this study. This software was divided into six stages of completion: patient's registration, symptoms, clinical and laboratory results, antibiogram, comorbidities, list of antibiotics. Data were collected prospectively, and patients were followed up until death or until hospital discharge. All data collected were confirmed by two specialists (José Victor Miranda Pedroso and Eduardo Leite Croco) and were cross-checked with laboratory information which was obtained directly from the laboratory’s database.

### Outcomes

The primary outcome was hospital mortality. The secondary outcome was an antimicrobial drug resistance. Antimicrobial drug resistance was defined as the ability of microorganisms, especially bacteria, to resist or to become tolerant to chemotherapeutic agents, antimicrobial agents, or antibiotics. This resistance may be acquired through gene mutation or foreign DNA in transmissible plasmids.

### Variables

The following data were collected prospectively for all included patients from the time of admission into the ICU: age, sex, length of hospital stay, the origin of infection, chronic diseases, and comorbidities, type of sepsis (sepsis, severe sepsis, and septic shock), need for mechanical ventilation, hemodialysis, surgical interventions, drainage, blood cultures, and antibiotic therapy. The severity of the disease was assessed using the APACHE II index (Acute Physiology And Chronic Health Evaluation II). The development of organ failures was accompanied by the SOFA index (Sequential Organ Failure Assessment). Both APACHE II index and SOFA were estimated on the first day of ICU admission and the last observation carried on before death or before ICU discharged.

### Statistical Analysis

Analyses were performed using Stata v.12 (Stata Corp., College Station, TX, United States). For descriptive statistics, we examined characteristics among patients who received EAT vs. patients who received therapy guided by blood culture. Based on distribution data we used unpaired *t*-test, chi-squared test, or Fisher's exact test.

The data were described using proportions and contingency tables for categorical variables and measures of central tendency and dispersion for continuous variables (age, Apache II, SOFA).

Hazard ratios (HRs) and 95% confidence intervals (CIs 95%) were obtained using Cox regression (proportional hazards regression) in order to estimate the effect of therapy directed by blood culture on mortality and hospital mortality. With regard to acquired antimicrobial drug multi-resistance, the relative risk and CIs 95% were calculated using Poisson regression. The statistical adjustment in all models included the following variables: sex, age, APACHE score, and SOFA score. All tests with *p*-values <0.05 were considered statistically significant.

## Results

**FIGURE 1:**
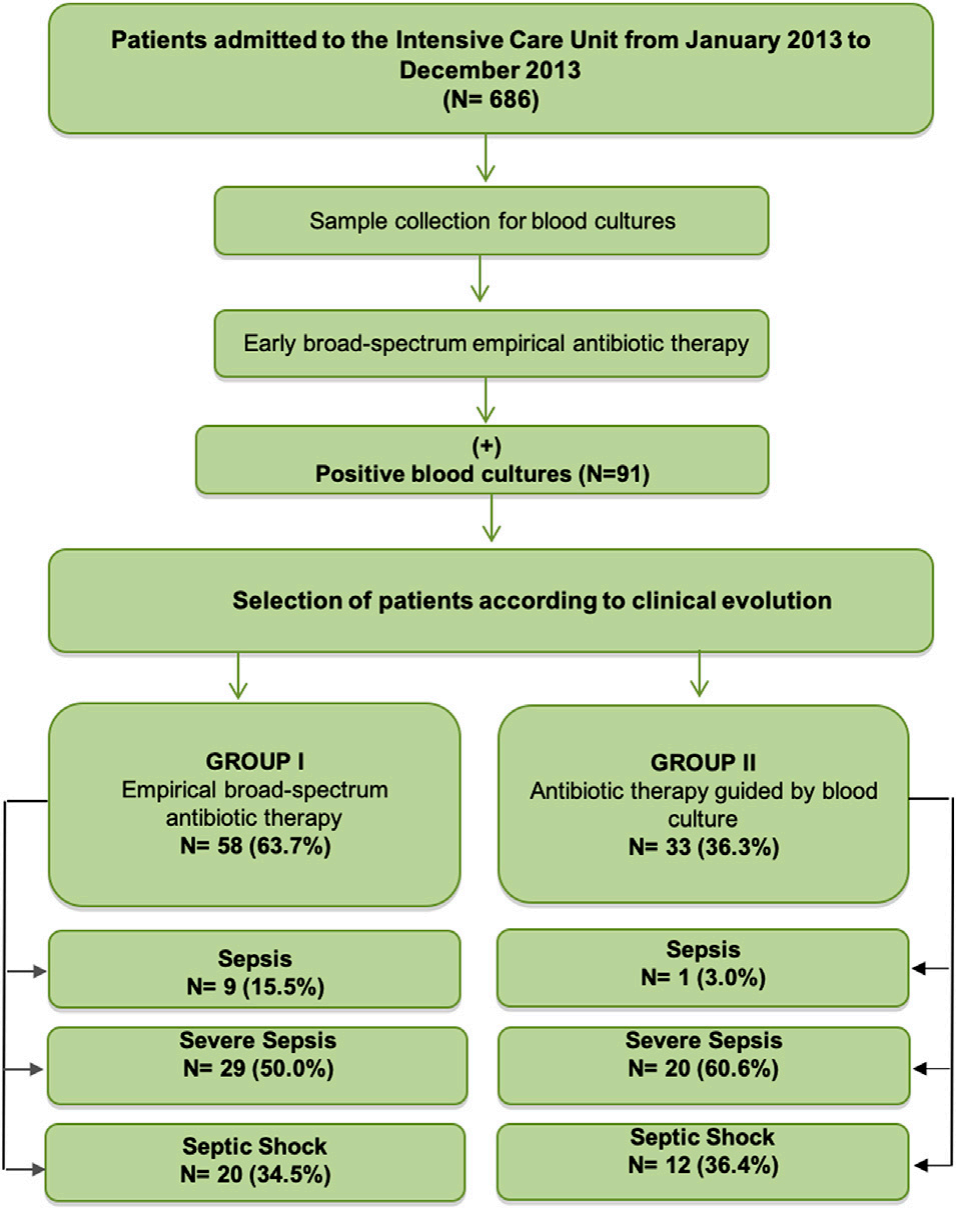
Study Flow diagram.

During the study period, 686 adults were admitted to the ICU, 91 eligible patients with sepsis, severe sepsis, or septic shock were included, according to the inclusion and exclusion criteria (Figure 1). The mean age of the patients was 52.7 years (standard deviation = 18.5 years) and 70.3% were male. They were classified into two groups: the broad-spectrum EAT group (58 patients) and the ATGBC group (33 patients).

Characteristics of the patients included in this study according to antibiotic therapy received are detailed in Table 1. There was no significant difference between patients who received EAT and the ATGBC with respect to sociodemographic characteristics such as age, sex, and ethnicity.

**TABLE 1:** Characteristics of patients with sepsis, severe sepsis or septic shock admitted to Intensive Care Unit (ICU) in a Public Hospital according to antibiotic therapy, Sorocaba city, State of São Paulo, Brazil, 2013 (*N* = 91).

	Empirical antibiotic therapy (EAT) *N* = 58 (63.7%)	Antibiotic therapy guided by blood culture (ATGBC) *N* = 33 (36.3%)	*p* > Value
Age (years)			0.68^a^
Mean ± sd	53.5 ± 17.7	51.9 ± 18.3	
Age group (years)			1.00^b^
≤65	41 (70.7)	23 (69.7)	
>65	17 (29.3)	10 (30.3)	
Sex			0.42^b^
Men	42 (72.4)	25 (75.7)	
Women	16 (27.6)	8 (24.2)	
Ethnicity			0.80^b^
Caucasian	44 (75.9)	28 (84.8)	
Black	14 (24.1)	5 (15.1)	
Severity			0.29^b^
Sepsis	9 (15.5)	1 (3.0)	
Severe sepsis	29 (50.0)	20 (60.6)	
Septic shock	20 (34.5)	12 (36.4)	
Comorbidities			
Presence of comorbidity	33 (56.9)	20 (60.6)	0.82^b^
Respiratory disease	4 (6.9)	1 (3.0)	1.00^b^
Cardiovascular disease	17 (29.3)	8 (13.8)	0.63^b^
Gastric disease	7 (12.0)	2 (6.0)	0.47^b^
Kidney disease	7 (12.0)	6 (18.2)	0.53^b^
Neurological disease	2 (3.5)	2 (6.0)	0.61^b^
Autoimmune disease	2 (3.5)	2 (6.0)	0.61^b^
Diabetes Mellitus	8 (13.8)	5 (15.2)	0.99^b^
Malignancy	5 (8.6)	2 (6.0)	1.00^b^
Coma on admission to the ICU	40 (68.9)	22 (66.6)	0.81^b^
Ventilator-associated pneumonia	13 (22.4)	11 (33.3)	0.54^b^
Apache II			0.83^a^
First day of ICU admission Mean ± sd	24.8 ± 7.4	24.1 ± 5.9	
Last observation^c^ Mean ± sd	20.6 ± 9.9	23.5 ± 6.8	
SOFA			0.71^a^
First day of ICU admission Mean ± sd	8.4 ± 3.7	7.5 ± 2.9	
Last observation^c^ Mean ± sd	5.6 ± 4.1	6.0 ± 3.2	
Length of hospitalization before the admission to ICU			0.44^a^
Mean ± sd	7.3 ± 10.9	5.6 ± 8.8	
Median (range)	2.5 (0 – 58)	2.0 (0 – 35)	

^a^ t test.

^b^ Fisher's exact test.

^c^ Last observation carried on before death or before ICU discharged.

Furthermore, statistically significant differences were not detected in the proportion of comorbidities, coma (*p* = 0.81), pneumonia associated with mechanical ventilation (*p* = 0.54) and in the median values of Apache II (*p* = 0.83) and SOFA (*p* = 0.71) and length of hospital stay before the ICU (*p* = 0.44) between the two groups.

The frequency of identified pathogens in the blood cultures according to antibiotic therapy received are presented in Table 2. Gram-positive was the most common in both types of antibiotic therapy (75.8% of patients with EAT vs. 87.9% of patients with ATGBC therapy, *p* = 0.18). *Staphylococcus* sp coag neg (63.6%) was the most frequently Gram-positive microorganism isolated, followed by *Enterococcus* spp. (34.6%). Among Gram-negative microorganisms, *Acinetobacter baumannii* (46.1%) was the most commonly isolated, followed by *Klebsiella pneumonia* (34.6%) and *Enterobacter erogenes* (34.6%).

**TABLE 2:** Microorganisms isolated in patients with sepsis. severe sepsis or septic shock obtained on admission to Intensive Care Unit (ICU) in a Public Hospital according to antibiotic therapy, Sorocaba city, State of São Paulo, Brazil, 2013 (*N* = 91).

Microorganisms	Empirical antibiotic therapy (EAT) *N* = 58		Antibiotic therapy guided by blood cultures (ATGBC) *N* = 33		*p* value
	Total	% MR^a^	Total	% MR^a^	
Gram-positive	44 (75.8)		29 (87.9)		0.18^b^
*Enterococcus* spp	9 (20.4)	1 (2.2)	7 (24.1)	1 (3.4)	
*Staphylococcus* sp coag neg	28 (63.6)	26 (59.0)	21 (72.4)	17 (58.6)	
*Staphylococcus aureus*	5 (11.3)	1 (2.2)	6 (20.6)	3 (10.3)	
*Streptococcus pneumoniae*	0 (0.0)	0 (0.0)	1 (3.4)	0 (0.0)	
Gram-negative	26 (44.8)		13 (39.4)		0.83^b^
*Enterobacter* aerogenes	9 (34.6)	4 (15.4)	3 (23.1)	2 (15.4)	
*Escherichia coli*	2 (7.7)	1 (3.8)	1 (7.7)	0 (0.0)	
*Klebsiella pneumoniae*	9 (34.6)	4 (15.3)	5 (38.4)	3 (23.0)	
*Morganella morganii*	1 (3.8)	0 (0.0)	1 (7.7)	0 (0.0)	
*Pseudomonas aeruginosa*	6 (23.0)	3 (11.5)	3 (23.0)	2 (15.3)	
*Serratia marcescens*	0 (0.0)	0 (0.0)	1 (7.7)	1 (7.7)	
*Acinetobacter* baumannii	12 (46.1)	11 (42.3)	6 (46.1)	6 (46.1)	
*Citrobacter freundii*	0 (0.0)	0 (0.0)	2 (15.3)	1 (7.7)	
*Citrobacter* koseri	1 (3.8)	0 (0.0)	0 (0.0)	0 (0.0)	
Burkholderia *cepacia*	0 (0.0)	0 (0.0)	1 (7.7)	0 (0.0)	

^a^ MR = Multi-resistance.

^b^ Fisher's exact test.

The most frequent multidrug-resistant bacteria isolated in critically ill patients were *Staphylococcus* sp, followed by *Acinetobacter baumanii* and *Klebsiella pneumoniae* (Table 3). Oxacillin-resistant *Staphylococcus* sp and Penicillin G-resistant *Staphylococcus* sp are the most commonly identified.

**TABLE 3:** The most common Multidrug Resistant Bacteria isolated in the blood cultures of patients with sepsis, severe sepsis or septic shock admitted to Intensive Care Unit (ICU) in a Public Hospital, Sorocaba city, State of São Paulo, Brazil, 2013 (*N* = 91).

	*Enterobacter* aerogenes			*Staphylococcus* sp coag neg		*Klebsiella pneumoniae*			*Pseudomonas aeruginosa*			*Acinetobacter* baumanii		
Antibiotic	Na	Rb (%)		Na	Rb (%)	Na	Rb (%)		Na	Rb (%)		Na	Rb (%)	
Penicillins														
Penicillin G	—	—		54	51 (94.4)	—	—		—	—		—	—	
Oxacillin	—	—		56	54 (96.4)	—	—		—	—		—	—	
Ampicillin	12	12 (100.0)		—	—	16	16 (100.0)		—	—		4	4 (100.0)	
Amoxicillin	10	10 (100.0)		—	—	14	9 (64.3)		—	—		—	—	
Piperacillin	13	11 (84.6)		—	—	16	11 (68.7)		11	7 (70.7)		18	17 (94.4)	
Cephalosporins														
1st Generation														
Cephalothin	12	12 (100.0)		—	—	15	11 (73.3)		—	—		—	—	
Cephalexin	—	—		—	—	—	—		—	—		—	—	
2nd Generation														
cefoxitin	12	12 (100.0)		—	—	17	12 (70.6)		—	—		—	—	
3rd Generation														
ceftazidime	12	10 (83.3)		—	—	15	10 (66.6)		—	—		—	—	
ceftriaxone	12	10 (83.3)				12	11 (91.6)		11	8 (72.7)		18	18 (100.0)	
4th Generation														
cefepime	12	10 (83.3)		—	—	16	11 (68.7)		11	8 (72.7)		17	16 (94.1)	
Carbapenems														
Meropenem	11	0 (0.0)		—	—	15	1 (11.2)		11	7 (63.6)		18	15 (83.3)	
Imipenem	12	0 (0.0)		—	—	15	0 (0.0)		11	7 (63.6)		18	14 (77.7)	
Ertapenem	12	1 (8.3)		—	—	15	0 (0.0)		—	—		—	—	
Quinolones														
Ciprofloxacin	12	6 (50.0)		51	45 (88.2)	16	10 (62.5)		11	8 (72.7)		18	16 (88.8)	
Aminoglycosides														
Amikacin	12	3 (25.0)		—	—	16	2 (12.5)		10	6 (60.0)		18	14 (77.7)	
Gentamycin	12	9 (75.0)		56	49 (87.5)	16	7 (43.8)		11	8 (72.7)		18	16 (88.8)	
Tobramycin	—	—		—	—	—	—		11	7 (63.6)		18	16 (88.8)	
Sulfonamides														
Sulfamethoxazole	11	7 (63.6)		54	44 (81.5)	16	10 (62.5)		—	—		18	16 (88.8)	
Macrolides														
Erythromycin	—	—		54	51 (94.4)	—	—		—	—		—	—	
Chloramphenicol														
Chloramphenicol	12	7 (58.3)		55	32 (58.2)	16	8 (50.0)		—	—		—	—	
Glycopeptides														
Vancomycin	—	—		54	1 (1.8)	—	—		—	—		—	—	
Lincosamides														
Clindamycin	—	—		52	48 (92.3)	—	—		—	—		—	—	
Monobactams														
Aztreonam	12	10 (83.3)		—	—	14	11 (78.6)		11	8 (72.7)		18	17 (94.4)	
Polymyxins														
Polymyxin B	—	—		—	—	—	—		10	1 (10.0)		18	1 (5.5)	
Oxazolidinones														
Linezolid	—	—		56	0 (0.0)	—	—		—	—		—	—	

^a^ N = number of positive blood cultures where specific antibiotic was tested.

^b^ R = number of positive blood cultures where the causative agent was resistant to the antibiotic.

Table 4 shows the antibiotics and number of days used according to intervention adopted. Piperacillin (36.2%), ceftriaxone (34.5%) and vancomycin (31.0%) were the antibiotics most used in EAT. About 90% of ATGBC patients used vancomycin, followed by piperacillin (51.5%) and cefepime (33.3%).

**TABLE 4:** Number of days of antibiotic use in patients with sepsis, severe sepsis or septic shock admitted to Intensive Care Unit (ICU) in a Public Hospital according to antibiotic therapy, Sorocaba city, State of São Paulo, Brazil, 2013 (N = 91).

Types of antibiotic	Empirical antibiotic therapy (EAT)		Antibiotic therapy guided by blood culture (ATGBC)	
	N = 58 (%)	Number of days Mean ± sd	N = 33 (%)	Number of days Mean ± sd
Penicillins	22 (37.9)		22 (66.6)	
Penicillin G	0 (0.0)	0.0 ± 0.0	1 (3.0)	8.0 ± 0.0
Oxacilin	1 (1.7)	12.0 ± 3.4	3 (9.1)	3.0 ± 1.5
Amoxicilin	0 (0.0)	0.0 ± 0.0	1 (3.3)	2.0 ± 0.0
Piperacilin + Tazobactam	21 (36.2)	10.4 ± 5.3	17 (51.5)	10.5 ± 3.5
Cephalosporins	39 (67,1)		24 (75.7)	
1st Generation				
Cefazolin	11 (18.9)	7.6 ± 4.2	3 (9.1)	1.6 ± 0.8
2nd Generation				
Cefuroxime	1 (1.7)	1.0 ± 0.0	0 (0.0)	0.0 ± 0.0
3rd Generation				
Ceftazidime	0 (0.0)	0.0 ± 0.0	1 (3.0)	4.9 ± 0.0
Ceftriaxone	20 (34.5)	8.15 ± 2.1	10 (30.3)	5.6 ± 2.5
4th Generation				
cefepime	7 (12.0)	7.1 ± 3.0	11 (33.3)	5.4 ± 3.5
Carbapenems	18 (31.0)		19 (57.6)	
Meropenem	9 (15.5)	15.2 ± 1.3	9 (27.3)	10.4 ± 4.1
Imipenem	5 (8.6)	10.6 ± 3.5	6 (18.2)	24.6 ± 2.0
Ertapenem	4 (6.8)	11.25 ± 2.8	4 (12.1)	8.5 ± 3.5
Quinolones	6 (10.3)		1 (3.0)	
Ciprofloxacin	5 (8.6)	7.0 ± 2.6	0 (0.0)	0.0 ± 0.0
Levofloxacin	0 (0.0)	0.0 ± 0.0	1 (3.0)	5.0 ± 0.0
Moxiflocin	1 (1.7)	3.0 ± 0.0	0 (0.0)	0.0 ± 0.0
Aminoglycosides	5 (8.6)		5 (15.2)	
Amikacin	4 (6.8)	7.5 ± 2.2	4 (12.1)	9.7 ± 1.5
Gentamycin	1 (1.7)	11.0 ± 0.0	1 (3.0)	21.0 ± 0.0
Sulfonamides	3 (5.2)		3 (9,1)	
Sulfamethoxazole	2 (3.4)	14.0 ± 0.0	2 (6.1)	3.0 ± 0.0
Sulfadiazine	1 (1.7)	7.0 ± 0.0	1 (3.0)	36.0 ± 0.0
Macrolides	2 (3.4)		2 (6.1)	
Rifampicin	2 (3.4)	17.5 ± 1.2	2 (6.1)	23.0 ± 0.0
Amphenicols	1 (1.7)		2 (6.1)	
Chloramphenicol	1 (1.7)	1.0 ± 0.0	2 (6.1)	0.0 ± 0.0
Glycylcyclines	1 (1.7)		1 (3.0)	
Tigecycline	1 (1.7)	19.0 ± 0.0	1 (3.0)	21.0 ± 0.0
Glycopeptides	19 (32.8)		30 (90.9)	
Vancomycin	18 (31.0)	13.6 ± 3.1	30 (90.9)	14.0 ± 5.2
Teicoplanin	1 (1.7)	1.0 ± 0.0	0 (0.0)	0.0 ± 0.0
Lincosamides	10 (17.2)		5 (15.2)	
Clindamycin	10 (17.2)	8.3 ± 2.4	5 (15.2)	11.0 ± 3.5
Polymyxins	3 (5.1)		7 (21.2)	
Polymyxin B	3 (5.1)	8.6 ± 1.2	7 (21.2)	9.8 ± 2.5
Oxazolidinones	1 (1.7)		3 (9.1)	
Linezolid	1 (1.7)	18.0 ± 0.0	3 (9.1)	12.0 ± 2.0

### Mortality

**FIGURE 2:**
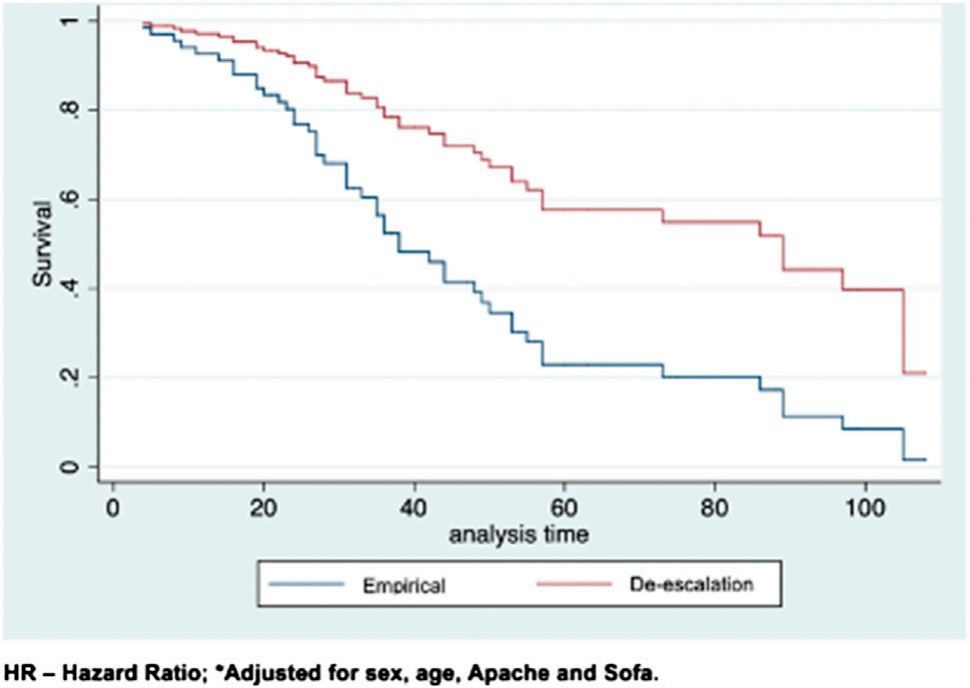
Survival in individuals with sepsis, severe sepsis or septic shock according to the length of hospital stay. Sorocaba city, State of São Paulo, Brazil 2013.

In the study sample of 91 individuals, 49 deaths were reported, resulting in a mortality rate of 53.8%. Mortality for patients who continued receiving EAT was 56.9% vs. 48.5% for patients receiving ATGBC. Figure 2 presents a survival analysis for patients during hospitalization. The hospital mortality rate of patients receiving ATGBC was statistically lower than the mortality rate of patients who continued receiving empirical treatment after adjustment for sex, age, APACHE II, and SOFA [HR 0.44 (IC 95% 0.24 – 0.82), *p* = 0.009] (Table 5).

**TABLE 5:** Mortality and Antimicrobial Drug Resistance (ADR) in *p* patients with sepsis, severe sepsis or septic shock admitted to Intensive Care Unit (ICU) in a Public Hospital according to antibiotic therapy, Sorocaba city, State of São Paulo, Brazil, 2013 (N = 91).

	Antibiotic therapy guided by blood cultures N (%)	Empirical antibiotic therapy (EAT) N (%)	Adjusted HR^a^ (95% CI^b^)	*p* value^c^
Mortality rate	16 (48.5)	28 (56.9)	0.44 (0.24–0.82)	0.009
Antimicrobial drug resistance	31 (93.9)	49 (84.5)	0.90 (0.78 – 1.03)	0.15

^a^ HR–Hazard Ratio.

^b^ CI–Confidence interval.

^c^ Ajusted for sex, age, Apache e Sofa.

### Antimicrobial Drug Resistance

The prevalence of ADR was high in both types of Antimicrobials therapy (93.5% ATGBC vs. 84.5% EAT). There was no association between ATGBC and ADR [HR 0.90 (95% CI 0.78 – 1.03) *p* = 0.15] (Table 5).

## Discussion

Although the safety and effectiveness of the strategy of de-escalation in the use of antibiotics in severe sepsis and septic shock were questioned in a previous systematic review (Silva et al., 2013), this prospective cohort study verified that the substitution of the EAT by an ATGBC increased survival of patients with sepsis when compared to the continuation of EAT, after adjusting for potentially confounding factors such as age, sex, APACHE. Currently, The Surviving Sepsis Campaign recommends that the antibiotic spectrum should be narrowed as soon as causative pathogens and their antibiotic susceptibility profile is available. In addition, some observational studies (Garnacho-Montero et al., 2014; Tabah et al., 2016) and a meta-analysis (Gutiérrez-Pizarraya et al., 2017) have also found that de-escalation therapy was associated with lower mortality rates in patients with severe sepsis and septic shock. It is important to highlight that although there is no consensus regarding the definition of de-escalation, switching from a broad to narrow therapy based on culture data constitutes one important strategy of this process (Tabah et al., 2016).

In this study, the alteration of initial empirical therapy was applied in one-third of patients. Although the severity of the disease did not influence the decision to use the ATGBC, some physicians may be reluctant to switch from a broad-spectrum antibiotic to narrow therapy when caring for patients with sepsis, especially when the infections are caused by multidrug-resistant bacteria. Furthermore, the applicability of antimicrobial therapy to infections caused by multidrug-resistant pathogens where the use of combined broad-spectrum therapy is recommended by most specialists, makes this choice very difficult to be adopted (Kollef et al., 2011).

Gram-positive bacteria were the most frequently involved microorganisms. These findings are consistent with the literature (Martin et al., 2003). *Staphylococcus* sp and *Acinetobacter baumannii* were the most prevalent multidrug-resistant bacteria. This result is similar to other studies about infections in Brazil (De Oliveira et al., 2012) and in other countries (Garnacho-Montero and Amaya-Villar, 2010; Teerawattanapong et al., 2018).

Despite the presence of ESKAPE organisms, the alteration of broad-spectrum empirical therapy by the narrow therapy did not influence the emergence of multidrug resistance pathogens. Although de-escalation is often presented as an effective strategy to reduce multidrug resistance, previous observational study (De Bus et al., 2016) and randomized clinical trial (Leone et al., 2014) found that the de-escalation did not affect the emergence of multidrug-resistant (MDR) pathogens. Only one study that evaluated the carbepen de-escalation has found significant differences in resistant *Acinetobacter* spp. colonization (Lew et al., 2015). According to De Waele et al. (2020) other studies with large numbers of patients are probably required to evaluate the impact of de-escalation on ADR in ICU patients.

In 2016, the new definitions of sepsis and septic shock have changed. Sepsis is now defined as life-threatening organ dysfunction caused by a dysregulated host response to infection. This definition uses the Sequential Organ Failure Assessment (SOFA ≥2) score to discriminate sepsis from uncomplicated infection, replacing SIRS criteria that were criticized for being inaccurate. SIRS criteria are present in many hospitalized patients, including those who never develop infection (Singer et al., 2016). Although our study followed the criteria established in 2001, all patients included in this study had SOFA ≥2. In addition, we only included patients with at least two positive blood cultures in order to ensure the diagnosis of sepsis in the patients included in this study.

### Limitations and Strength of This Study

To our knowledge, this study adds valuable information on the management of antibiotic therapy in patients with sepsis since there are few data or recommendations that assess effective approaches to sepsis treatment in resource-limited low-income and middle-income countries such as Brazil. The development of local studies that analyze the use of antimicrobials, as well as ADR in hospital settings, is important since the causes of sepsis and available infrastructure can be different from those described in high-income countries. It is also important to highlight that this research included the application of a robust methodology. During the entire study period, the authors made sure that the initial empirical therapy and their alteration were based on protocol established by Hospital Infection Control Committee (HICC) which monitor the use of antibiotics and resistant strains in the hospital. The local policy regarding empiric therapy was based on antimicrobial resistant patterns present in the hospital and followed the Surviving Sepsis Campaign guideline (Dellinger et al., 2013) and used the criteria of evolutionary clinicians according to Bone et al. (1992) *.* Furthermore, this study compared two types of therapies using robust outcome measures (mortality of hospital and antimicrobial drug resistance). Data collection was prospective and cross-checked with laboratory information which was obtained directly from the laboratory's database. In addition, there was no loss of patients in this sample, all the results collected were analyzed within the original patient group and the outcomes were adjusted by confusing factors such as sex, age, APACHE II, and SOFA.

However, some limitations of our study should be acknowledged. First, the de-escalation practice was based on physician preference. Second, this study involved a small number of patients from a single public hospital. In addition, although the all patients included in this study have the possibility of de-escalation, the number of patients who had alteration of initial empirical therapy was limited. It is important to emphasize that this work was a cohort study, patients were not randomised to the two options. Thus, the de-escalation was based on clinical decision making.

Third, we initially used medical records to collect data from patients which these are not designed for research purposes. Thus, in order to reduce memory bias or lack of data in medical records any question or absence of data was requested to the team in real-time, and all variables collected were confirmed by two specialists. Finally, only patients with positive blood cultures were included in order to reduce the colonization/contamination bias due to the presence of bacteria in catheters and other access points in patients. Despite the blood culture remains the reference standard for the diagnosis of sepsis, contaminations represent up to 50% of positive blood cultures (Dargère et al., 2018). This limitation implied that, for all patients, the possibility of de-escalation existed. On the other hand, this inclusion criterion increases the specificity of the diagnosis of sepsis since the SIRS has limited specificity.

### Implications for Clinical Practice

In order to improve antibiotic prescribing patterns, it is well established that accurate knowledge of the pathogens associated, and their susceptibility profile allows for a more rational selection of antibiotics for treatment of patients with sepsis. In clinical practice, however, antibiotics are often used, even when culture results are not available. The challenge for intensive care physicians is to obtain microbiological cultures before starting antimicrobial therapy (Schurink et al., 2004).

Although the early and appropriate EAT is undoubtedly an important prognostic factor in critically ill patients with sepsis, our findings clearly support the use of empirical antibiotics should be reviewed according to the susceptibility profile, when blood culture results are available. Thus, the ideal approach for the treatment of patients with sepsis, severe sepsis and septic shock admitted to the ICU should include the early administration of broad-spectrum antibiotics, together with reassessment and narrowing or discontinuation of subsequent treatment based on the results of blood cultures and antibacterial susceptibility. However, further studies are needed in order to evaluate the impact of the implementation of this practice on antibiotic resistance.

On the other hand, it is important to highlight that routine microbiological cultures and sensitivity tests may not be performed in some hospitals from most low- and middle-income countries, such as Brazil, due to a lack of personnel trained, equipment and financial resources (Taniguchi et al., 2019; Kakkar et al., 2020; Mathew et al., 2020). Without this information, clinicians do not have sufficient information to prescribe the narrowest-spectrum antibiotic needed to treat patients with sepsis. Therefore, the transferability of our findings to hospitals with more limited resources within Brazil or to other low- and middle-income countries will largely depend on the availability of laboratory support and financial resources.

## Data Availability

The raw data supporting the conclusions of this article will be made available by the authors, without undue reservation.
